# Pharmacokinetic/pharmacodynamic parameters of vancomycin for predicting clinical outcome of enterococcal bacteremia

**DOI:** 10.1186/s12879-022-07668-w

**Published:** 2022-08-10

**Authors:** Eliel Nham, Kyungmin Huh, You Min Sohn, Hyo Jung Park, Hyemee Kim, Sook Young Woo, Jae-Hoon Ko, Sun Young Cho, Cheol-In Kang, Doo Ryeon Chung, Hee Jae Huh, Hyung-Doo Park, Nam Yong Lee, Kyong Ran Peck

**Affiliations:** 1 Division of Infectious Diseases, Department of Medicine, Korea University School of Medicine, Seoul, South Korea; 2 Division of Infectious Diseases, Department of Medicine, Samsung Medical Center, Sungkyunkwan University School of Medicine, 81 Irwon-ro, Gangnam-gu, Seoul, 06351 South Korea; 3 Department of Pharmaceutical Services, Samsung Medical Center, School of Pharmacy, Sungkyunkwan University, Seoul, South Korea; 4 Statistics and Data Center, Samsung Medical Center, Seoul, South Korea; 5 Department of Laboratory Medicine and Genetics, Samsung Medical Center, Sungkyunkwan University School of Medicine, Seoul, South Korea

**Keywords:** Enterococcus, Vancomycin, PK/PD, AUC/MIC, Trough

## Abstract

**Purpose:**

To find pharmacokinetic/pharmacodynamic parameters of vancomycin associated with the optimal outcome of severe infection due to *Enterococcus* species.

**Methods:**

We retrospectively reviewed enterococcal bacteremia cases treated with vancomycin from January 2015 to December 2020. The primary outcome was 30-day mortality. We calculated cutoff values of the ratio of vancomycin area under the concentration–time curve over 24 h to the minimum inhibitory concentration (AUC_24_/MIC) and trough concentration (C_trough_) during the initial 72 h of treatment. The optimal cutoff value was determined using the Youden index. Binary variables created based on these cutoffs were further assessed using multivariable analysis.

**Results:**

A total of 65 patients were included. The majority (87.7%) had solid or hematologic malignancies. Thirty-day mortality and nephrotoxicity occurred in nine (13.4%) and 14 (21.5%) patients, respectively. Both vancomycin AUC_24_/MIC and C_trough_ showed fair performance in predicting 30-day mortality (AUC of receiver-operator curve for AUC_24_/MIC, 0.712; 95% confidence interval [CI] 0.539–0.886; AUC for C_trough_, 0.760; 95% CI 0.627–0.892; pairwise AUC comparison: p = 0.570). C_trough_ ≥ 13.94 μg/mL, but not AUC_24_/MIC ≥ 504, had a significant association with 30-day mortality after adjusting for confounders (odds ratio, 8.40; 95% CI 1.60–86.62; p = 0.010).

**Conclusion:**

Mean C_trough_ ≥ 13.94 μg/mL during the initial 72 h was associated with higher 30-day mortality in enterococcal bacteremia. Further studies are warranted to elucidate optimal pharmacokinetic targets for enterococcal bacteremia.

**Supplementary Information:**

The online version contains supplementary material available at 10.1186/s12879-022-07668-w.

## Introduction

*Enterococcus* spp. are some of the common causes of nosocomial infections, and their significance has increased in the last several decades [1, 2]. Enterococcal infections are often treated with vancomycin when isolates are resistant to penicillin but susceptible to glycopeptides. Patients most affected by invasive enterococcal infections are those with multiple comorbidities. These patients are also more susceptible to nephrotoxicity [3]. Therefore, it is crucial to optimize vancomycin treatment for invasive enterococcal infections. Data regarding optimal pharmacokinetic/pharmacodynamic (PK/PD) parameters of vancomycin for serious infections caused by gram-positive organisms other than methicillin-resistant *Staphylococcus aureus* (MRSA) are scarce. Guidelines for therapeutic monitoring of vancomycin by the American Society of Health-System Pharmacists (ASHP) have confined their recommendations of its use to serious MRSA infections [4].

There are three small retrospective studies regarding this topic. One of these studies only assessed trough concentrations, a surrogate marker of the area under the curve (AUC) of the concentration–time curve [5–7]. A recently published larger retrospective study was limited by the inclusion of a considerable number of non-severe infections [8]. A prospective study from China also attempted to provide insights into this matter [9]. This study included both staphylococcal and enterococcal infections. However, the latter comprised only 23.19% of total infections, making it difficult to generalize the study results to enterococcal infections. Therefore, we aimed to determine the PK/PD parameters associated with the optimal clinical outcomes of enterococcal bacteremia.

## Methods

### Study population

We retrospectively reviewed all cases of enterococcal bacteremia from January 2015 to December 2020 at the Samsung Medical Center, a 1950-bed tertiary referral center in South Korea. Patients with enterococcal bacteremia who received intravenous vancomycin for an initial 72 h or longer were included. Patients whose isolates were resistant to vancomycin, who did not have vancomycin concentrations measured, received concurrent antimicrobial which is active against the isolated enterococci, were receiving renal replacement therapy, had polymicrobial bacteremia, or for whom treatment was considered futile due to underlying disease were excluded. If a patient had more than one episode of enterococcal bacteremia within the study period, only bacteremic episodes with a minimum of a 1-year interval since the last episode were included.

### Study design and definitions

We selected two PK/PD parameters: the ratio of vancomycin area under the concentration–time curve over 24 h to the minimum inhibitory concentration (AUC_24_/MIC) and the trough concentration (C_trough_). The primary outcome was 30-day all-cause mortality. Secondary outcomes were clinical failure at the end of treatment (EOT), the incidence of nephrotoxicity, and 90-day recurrence. Clinical failure was defined as any of the following: presence of fever, hemodynamic instability, or death. Cutoff values of the PK/PD parameters for prediction of the primary outcome were determined from the receiver operating characteristic (ROC) curve. The study subjects were then divided based on these cutoff values. Baseline clinical characteristics and clinical outcome measures were compared between groups. Finally, independent risk factors for 30-day mortality were elucidated using a logistic regression model, including the PK/PD parameters.

Patients were considered immunocompromised if they had been on steroids equivalent to ≥ 20 mg/day of prednisolone for ≥ 3 weeks, received other immunosuppressants in the absence of active malignancy, or received a solid organ or hematopoietic stem cell transplant. Primary bacteremia was defined as bloodstream infection without an identifiable source of infection. The day of the first positive blood culture was defined as day 1. Acute kidney injury (AKI) [10], chronic kidney disease [11], Charlson comorbidity index (CCI) [12] and the Pitt bacteremia score [13] were defined as per previous studies. Creatinine clearance (CL_Cr_) was calculated using the Cockcroft–Gault formula (14). In line with previous literature, nephrotoxicity was defined as an increase of > 0.5 mg/dL or a ≥ 50% increase in serum creatinine over baseline or a decrease in calculated CL_Cr_ of 50% from baseline on 2 consecutive days [4].

### Microbiologic and pharmacokinetic/pharmacodynamic analysis

Species identification and antimicrobial susceptibility testing were performed using the VITEK 2 system (bioMérieux, Marcy-l’Etoile, France). MICs were then confirmed by the broth microdilution (BMD) method according to the Clinical and Laboratory Standards Institute guidelines [15, 16].

The institutional protocol states that vancomycin is infused over an hour if the dose is ≤ 1 g or 2 h if > 1 g. Blood samples were drawn immediately before the administration of the fourth dose for C_trough_ calculation. Since there is no established PK target for enterococcal bacteremia, clinicians adjusted vancomycin doses at their discretion, guided by the C_trough_. Initial doses were given at 12-h intervals. The dosing interval was then adjusted according to the trough concentrations. From April 2017, loading doses of 25 mg/kg were administered to patients with hematologic malignancies or critical illnesses. The serum vancomycin concentration was measured using a fluorescence polarization immunoassay (COBAS INTEGRA Vancomycin, Roche, Germany).

AUC_24_ was calculated using the Bayesian method with the APK© software (version 3.5.28, RxKinetics, Plattsburg, MO). As previous studies suggested that the vancomycin AUC/MIC be optimized early in the course of infection [17–19], AUC_24_/MIC and C_trough_ during the initial 72 h of treatment were chosen as measures of drug exposure. The average AUC_24_/MIC and C_trough_ values during the initial 72 h were calculated as arithmetic means.

### Statistical analysis

Cutoff values of AUC_24_/MIC and C_trough_ were determined using the Youden index [20]. Continuous variables were compared using Student’s *t*-test or the Mann–Whitney *U* test. Categorical variables were compared using χ^2^ or Fisher’s exact test. Variables with a *p*-value < 0.1 were further examined by logistic regression with backward selection. Firth’s penalized-likelihood logistic regression was performed to resolve the complete separation of some variables (e.g., no one in the treatment or control group experienced the outcome event) [21]. Statistical significance was defined as a two-sided *p*-value < 0.05. All statistical analyses were executed by R (version 4.1.1, R Foundation for Statistical Computing, Vienna, Austria).

## Results

### Baseline characteristics

A total of 65 patients were included in this study. The majority of the study population were elderly men with normal renal function and a high burden of comorbidity (Table 1). The most common cause of bacteremia was intraabdominal infection (50.8%), followed by primary bacteremia (35.4%). The median Pitt score was 0 (interquartile range [IQR], 0–2), and 3.1% of the population required ICU care at the onset of bacteremia. Thirty-day mortality and nephrotoxicity occurred in nine (13.8%) and 14 (21.5%) patients, respectively. The median time to the incidence of nephrotoxicity was 11 days (IQR, 7–15).

**Table 1:** Baseline characteristics of study subjects (n = 65)

Characteristic	Number (%) or median (IQR)
Demographics	
Age (years) (mean, SD)	60 (14)
Male sex	46 (70.8%)
Body mass index (kg/m^2^) (mean, SD)	22.2 (3.1)
Comorbidities	
Diabetes mellitus	8 (12.3%)
Liver cirrhosis	5 (7.7%)
Chronic kidney disease	0 (0.0%)
Solid organ transplant	8 (12.3%)
Solid cancer	29 (44.6%)
Hematologic cancer	28 (43.1%)
Immunocompromised	1 (1.5%)
Charlson comorbidity index	5 (3, 7)
Source of bacteremia	
Intraabdominal infection	33 (50.8%)
Urinary tract infection	4 (6.2%)
Primary bacteremia	23 (35.4%)
Others	5 (7.7%)
Severity of infection	
Pitt bacteremia score	0 (0, 2)
ICU admission at the onset of bacteremia (n = 63*)	2 (3.1%)
Acute kidney injury at the onset of bacteremia	8 (12.3%)
Laboratory test at the onset of bacteremia	
White blood cell count (× 10^3^/μL)	5.83 (0.28, 12.03)
Neutropenia	21 (32.3%)
C-reactive protein (mg/dL) (n = 64**)	8.9 (5.4, 12.9)
CL_Cr_ (mL/min)	96.7 (59.8, 123.9)
Microbiologic test results	
* Enterococcus faecium* isolated	62 (95.4%)
Vancomycin MIC determined by broth microdilution (μg/mL)	1 (1, 1)
Factors related to vancomycin treatment	
Initiation of vancomycin as empiric antibiotic	62 (95.4%)
Use of loading dose	14 (21.4%)
Average AUC_24_ during initial 72 h (mg/L)	555 (443, 621)
Average AUC_24_/MIC during initial 72 h	579 (453, 676)
Average trough concentration (μg/mL) (mean, SD)	13.71 (4.48)
Duration of treatment (days)	10 (7, 13)
Other factors potentially related to prognosis	
Bloodstream infection with other microorganism(s) within a month	27 (41.5%)
Source control indicated	26 (40.0%)
Source control performed (n = 26)	12 (18.5%)
Infectious diseases consultation	45 (69.2%)

IQR, interquartile range; SD, standard deviation; ICU, intensive care unit; CL_Cr_, creatinine clearance; MIC, minimum inhibitory concentration; AUC_24_, area under the curve during 24 h; N/A, not applicable

*Two patients who were already in the ICU at the onset of bacteremia were not included

**One patient who did not have C-reactive protein measured at the onset of bacteremia was not included

Sixty-two (95.4%) of the isolates were *Enterococcus faecium*, and 84.6% of isolates had MIC values measured by a BMD of 1 μg/mL. A considerable discrepancy was noted between the MIC measured by automated BMD (MIC_autoBMD_) and manual BMD (MIC_BMD_). Among the 53 isolates with MIC_autoBMD_ ≤ 0.5 μg/mL, 46 (86.8%) had a MIC_BMD_ of 1 μg/mL. In addition, two (25%) out of eight isolates with a MIC_autoBMD_ of 1 μg/mL had an MIC_BMD_ of 2 μg/mL. The MIC_BMD_ was used to calculate the AUC_24_/MIC throughout the study. The median value of average AUC_24_/MIC during the initial 72 h was 579 (IQR, 453–676), and the mean C_trough_ was 13.71 μg/mL (standard deviation, 4.48). The average AUC_24_/MIC and C_trough_ values showed a moderate correlation (Spearman’s rank correlation coefficient = 0.518, *p* < 0.001).

### Determining PK/PD parameters for predicting clinical outcomes

**Fig. 1:**
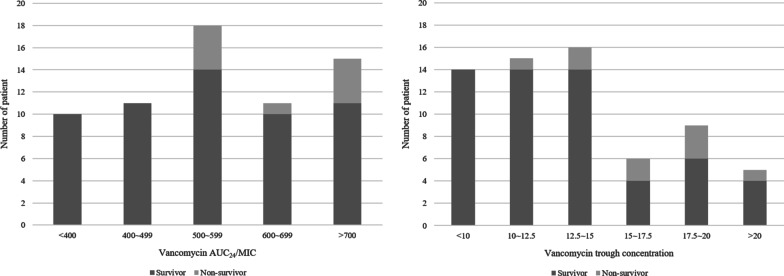
Distribution of 30-day mortality across vancomycin AUC_24_/MIC and trough concentrations. MIC, minimum inhibitory concentration; AUC_24_, area under the curve during 24 h

The distribution of 30-day mortality across vancomycin AUC_24_/MIC and C_trough_ during the initial 72 h is presented in Fig. 1. No patient with an average AUC_24_/MIC < 500 died within 30 days after the onset of bacteremia. In addition, all patients with an average vancomycin C_trough_ < 10 μg/mL survived by 30 days.

**Fig. 2:**
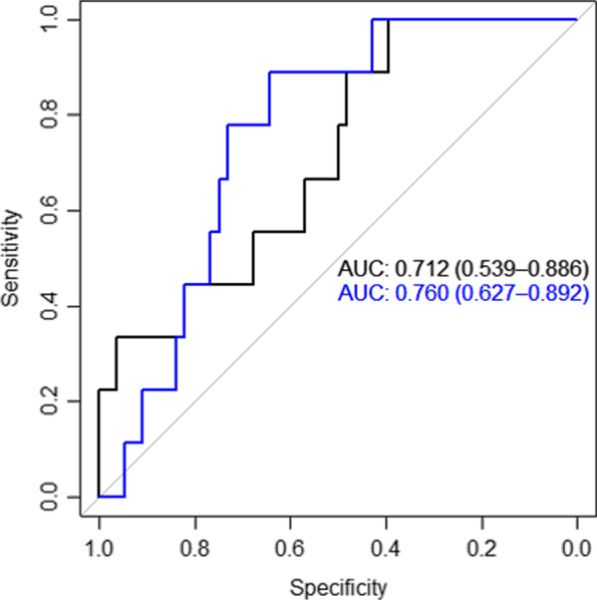
Receiver operating characteristic curve for the prediction of 30-day mortality by AUC_24_/MIC and trough concentration (black line: AUC_24_/MIC, blue line: trough concentration). MIC, minimum inhibitory concentration; AUC_24_, area under the curve during 24 h

The cutoff value of the average AUC_24_/MIC during the initial 72 h was 504 for prediction of 30-day mortality. The AUC of the ROC curve was 0.712 (95% confidence interval [CI] 0.539–0.886; Fig. 2). The cutoff value of the average C_trough_ during the initial 72 h was 13.94 μg/mL and that of the AUC was 0.760 (95% CI 0.627–0.892). As expected from the distribution presented in Fig. 1, these cutoff values were notable for their high sensitivity (1.000) and low specificity (0.379). There was no significant difference between AUC_24_/MIC and C_trough_ in terms of predictive performance (pairwise comparison of AUC curves: p = 0.570).

When the study population was divided into two groups based on each cutoff value, there was no difference in baseline characteristics between the groups. This was except for a higher proportion of urinary tract infections and lower CL_Cr_ in the C_trough_ ≥ 13.94 μg/mL group (Additional file 1: Tables S1 and S2).

Thirty-day mortality occurred exclusively in the high AUC_24_/MIC group (0.0% vs. 20.9%, p = 0.023; Table 2). Nephrotoxicity occurred more frequently in patients with C_trough_ ≥ 13.94 μg/mL (8.1% vs. 39.3%, p = 0.022). The proportion of patients who experienced clinical failure at EOT or 90-day recurrence did not differ between the two groups.

**Table 2:** Incidence of primary and secondary outcomes in groups divided based on cutoff values

	Trough < 13.94 μg/mL (n = 37)	Trough ≥ 13.94 μg/mL (n = 28)	Odds ratio	*p*-value
30-day mortality	1 (2.7%)	8 (28.6%)	14.40 (1.68–123.56)	0.004
Treatment failure at EOT (n = 64)*	7 (19.4%)	8 (28.6%)	1.65 (0.51–5.30)	0.553
Nephrotoxicity (n = 63)**	3 (8.1%)	11 (39.3%)	7.33 (1.80–29.83)	0.005
90-day recurrence (n = 64)***	4 (10.8%)	3 (11.1%)	1.08 (0.22–5.27)	> 0.999

	AUC_24_/MIC < 504 (n = 22)	AUC_24_/MIC ≥ 504 (n = 43)	Odds ratio	*p*-value
30-day mortality	0 (0.0%)	9 (20.9%)	N/A	0.023
Treatment failure at EOT (n = 64)*	3 (13.6%)	12 (27.9%)	2.23 (0.58–9.34)	0.348
Nephrotoxicity (n = 63)**	2 (9.1%)	12 (27.9%)	3.87 (0.78–19.15)	0.114
90-day recurrence (n = 64)***	2 (9.1%)	5 (11.9%)	1.39 (0.25–7.82)	> 0.999

EOT, end of treatment; ICU, intensive care unit; N/A: not applicable; MIC, minimum inhibitory concentration; AUC_24_, area under the curve during 24 h

*One patient whose treatment outcome was not assessable for having been referred to another hospital for the remaining treatment was excluded

**Two patients who were already in the ICU at the onset of bacteremia were not included

***One patient who died within 90 days without clearance of bacteremia was not included

Even though short-term mortality is a preferred measure of treatment outcome due to its objectivity, one may argue that mortality after enterococcal bacteremia cannot be attributed to the infection in considerable cases. Therefore, we attempted to build another prediction model for clinical failure at EOT. However, the models had no ability to predict clinical failure, as the 95% CI of the AUC of each ROC curve included 0.5 (for AUC_24_/MIC, AUC 0.536 [95% CI 0.387–0.685]; for C_trough_, AUC 0.561 [95% CI 0.397–0.724]).

### Risk factors for 30-day mortality

Patients with higher average AUC_24_/MIC and C_trough_ and those with higher white blood cell counts were more likely to die within 30 days after bacteremia (Table 3). Diabetes and AKI at the onset of bacteremia were marginally associated with 30-day mortality. Among these characteristics, vancomycin C_trough_ ≥ 13.94 μg/mL was the only statistically significant risk factor for mortality in a multivariable model (OR 8.40, 95% CI 1.60–86.62, p = 0.010).

**Table 3:** Comparison of characteristics by 30-day mortality

	Survival (n = 56)	Death (n = 9)	Odds ratio (95% CI)	*p*-value	Adjusted odds ratio (95% CI)	*p*-value
Demographics						
Age (year)	59 (53, 70)	64 (52, 71)		0.864		
Male sex	39 (69.6%)	7 (77.8%)	1.53 (0.29–8.12)	> 0.999		
Body mass index (kg/m^2^)	22.1 (20.1, 24.1)	22.4 (18.7, 23.3)		0.488		
Comorbidities						
Diabetes mellitus	5 (8.9%)	3 (33.3%)	5.10 (0.97–26.89)	0.074	5.58 (0.90–38.55)	0.064
Liver cirrhosis	5 (8.9%)	0 (0.0%)	N/A	> 0.999		
Solid organ transplant	7 (12.5%)	1 (11.1%)	0.88 (0.09–8.09)	> 0.999		
Solid cancer	33 (58.9%)	3 (33.3%)	0.35 (0.07–1.54)	0.278		
Hematologic cancer	26 (46.4%)	2 (22.2%)	0.33 (0.06–1.73)	0.280		
Charlson comorbidity index	5 (3, 6)	8 (3, 9)		0.276		
Source of bacteremia						
Intraabdominal infection	29 (51.8%)	4 (44.4%)	1.34 (0.33–5.53)	0.733		
Urinary tract infection	4 (7.1%)	0 (0.0%)	N/A	> 0.999		
Primary bacteremia	19 (33.9%)	4 (44.4%)	1.56 (0.37–6.49)	0.709		
Others	4 (7.1%)	1 (11.1%)	1.63 (0.16–16.44)	0.536		
Severity of infection						
Pitt bacteremia score	0 (0, 1)	2 (0, 2)		0.134		
ICU admission at the onset of bacteremia	2 (3.7%)	0 (0.0%)	N/A	> 0.999		
AKI at the onset of bacteremia	5 (8.9%)	3 (33.3%)	5.10 (0.97–26.89)	0.074		
Laboratory test results						
Neutropenia	21 (37.5%)	0 (0.0%)	N/A	0.026		
White blood cell count (× 10^3^/μL)	3.00 (0.22, 10.31)	11.08 (7.97, 13.68)		0.024	1.04 (1.00–1.16)	0.072
C-reactive protein (mg/dL)	8.9 (5.4, 12.9)	8.9 (4.9, 12.5)		0.643		
Creatinine clearance (mL/min)	96.4 (64.8, 125.6)	98.6 (57.8, 106.4)		0.857		
Factors related to vancomycin treatment						
Use of vancomycin loading dose	44 (78.6%)	7 (77.8%)	0.95 (0.18–5.21)	> 0.999		
Average AUC_24_ during initial 72 h (mg/L)	531 (435, 603)	616 (562, 706)		0.016		
Average AUC_24_/MIC during initial 72 h	564 (435, 658)	616 (561, 1053)		0.043		
Average AUC_24_/MIC ≥ 504	34 (60.7%)	9 (100.0%)		0.023		
Average trough during initial 72 h (μg/mL)	12.52 (10.14, 15.14)	15.88 (14.94, 17.86)		0.013		
Average trough ≥ 13.94 μg/mL	20 (35.7%)	8 (88.9%)	14.40 (1.68–123.56)	0.004	8.40 (1.60–86.62)	0.010
Duration of vancomycin treatment (days)	11 (8, 14)	7 (7, 10)		0.034		
Nephrotoxicity	10 (17.9%)	4 (44.4%)	3.68 (0.84–16.20)	0.091		
Other factors potentially related to prognosis						
Bloodstream infection due to other microorganism(s) within a month	24 (42.9%)	3 (33.3%)	0.67 (0.15–2.94)	0.724		
Source control indicated	21 (37.5%)	5 (55.6%)	2.08 (0.50–8.63)	0.465		
Source control performed (n = 26)	10 (47.6%)	2 (40.0%)	0.73 (0.10–5.33)	> 0.999		
Consult with infectious disease expert	17 (30.4%)	3 (33.3%)	1.15 (0.26–5.13)	> 0.999		

Logistic regression with backward selection (threshold *p* < 0.10) was used for the multivariable model

CI, confidence interval; ICU, intensive care unit; AKI, acute kidney injury; AUC_24_, area under the curve during 24 h; MIC, minimum inhibitory concentration; N/A, not applicable

*Amikacin was the only aminoglycoside in all patients who received aminoglycoside concurrently with vancomycin

## Discussion

We found that both vancomycin AUC_24_/MIC and C_trough_ during the initial 72 h of treatment had fair performance in predicting 30-day mortality. However, when AUC_24_/MIC and C_trough_ were transformed into binary variables with each cutoff value and analyzed in the regression model, only C_trough_ ≥ 13.94 μg/mL was an independent risk factor for 30-day mortality in patients with enterococcal bacteremia.

The literature suggests that the risk of nephrotoxicity increases as a function of C_trough_ or AUC [22–24], which is in accordance with our results. In our study, a higher C_trough_ was significantly associated with both 30-day mortality and nephrotoxicity, but not with clinical failure. This may be explained by a higher burden of comorbidity and a higher incidence of both AKI at the onset of bacteremia and nephrotoxicity in those who died within 30 days. Although these variables turned out to be statistically insignificant after multivariable analysis, it is possible that we did not find a significant association due to the small population size.

Three studies demonstrated the benefit of higher vancomycin exposure in the treatment of enterococcal infections, all of which were notably different from our study [5, 7, 8]. In studies by Jumah et al. [5] and Sohn et al. [7], the severity of infection measured by the proportion of ICU patients at baseline, who might benefit from higher drug exposure, was higher than that of our population (14% and 37.8%, respectively, compared to 3.1% in our study). Moreover, those studies included larger proportions of infection with *Enterococcus faecalis* (36.8% and 29.7%, respectively, compared to 4.5% in our study), the majority of which are susceptible to penicillin. Although the proportion of concurrent antibiotic use did not differ between the survivor and non-survivor groups in those studies, there might have been an unadjusted difference in the use of antibiotics that are active against enterococci. Considering the low Pitt bacteremia score and low percentage of ICU admissions in our study, avoiding an unnecessarily high C_trough_ might be justifiable when treating infections in non-severe conditions. Katip et al. restricted their study population to ampicillin-resistant enterococcal infections and reported that vancomycin AUC_24_/MIC ≥ 400 during the initial 72 h of treatment was associated with a higher likelihood of a better clinical outcome, which was a composite of the resolution of signs and symptoms related to enterococcal infection [8]. However, the adjusted hazard ratio for nephrotoxicity was 3.95 (95% CI 1.09–14.47). This might be quite high considering that the study population largely comprised patients with non-severe infections (non-bacteremic urinary tract infections, 70.2%; non-bacteremic wound infections, 12.5%).

C_trough_ has been used as a surrogate marker for AUC/MIC because it is difficult to estimate the AUC using conventional methods. The new ASHP guidelines updated in 2020 no longer recommend C_trough_-based monitoring for serious MRSA infections [4]. However, they acknowledged that there is insufficient evidence to apply the same recommendation to non-MRSA infections. In addition to our study, Nakakura et al. [6] and Sohn et al. [7] found a significant association between C_trough_ and mortality. Considering this, clinicians who practice in settings where AUC_24_/MIC are not routinely obtained may benefit from monitoring C_trough_ when treating enterococcal bacteremia.

Our study has several strengths. First, by measuring MICs with BMD, the reliability of AUC_24_/MIC was higher than in previous studies, all of which did not confirm MIC by BMD or the E-test except Jumah et al. [5]. Second, we excluded cases in which antibiotics other than vancomycin with in vitro activity against enterococci were used.

However, this study also has important limitations. First, there are inherent limitations from the retrospective nature of the study. We used logistic regression to mitigate confounding; however, the possibility of unobserved bias could not be excluded. Furthermore, there might have been variability in how vancomycin dosage was adjusted after the measurement of C_trough_, which is difficult to control in a retrospective study and could have affected treatment outcomes. Second, the composition of the patients included in our study may limit the generalizability of the results. Patients with chronic kidney disease were unintentionally excluded due to the exclusion criteria unrelated to renal function. Also, most of the patients had either solid or hematological malignancies. Third, intraabdominal infection was the focus of bacteremia in about half of the study population. Considering the often polymicrobial nature of intraabdominal infections, antimicrobial treatment other than vancomycin might have affected the clinical outcomes. However, we excluded the patients with polymicrobial bacteremia to minimize such impact. Fourth, we calculated AUC_24_/MIC with a single concentration (C_trough_) since peak concentrations had not been measured. However, while larger studies should verify it, Neely et al. reported that trough-only data could generate reliable AUC estimates [25]. Fifth, there are limitations in the outcome measures. Although mortality is generally the most objective indicator, it may not fully reflect the clinical outcome when infection-attributable mortality is low. Clinical failure, one of the secondary outcomes, included some subjective variables. However, higher C_trough_ was consistently not associated with better clinical outcomes in various measures. Last, there is a risk of overfitting. The study dataset was used both for derivation and application of the ROC curves. Furthermore, there is a sampling error in the selection of thresholds. The result from this study needs external validation in future studies. Our results call for a prospective study with a larger population to determine PK parameters to optimize outcomes in enterococcal bacteremia.

In conclusion, monitoring C_trough_ during the initial 72 h may be useful as a pharmacokinetic target for the treatment of enterococcal bacteremia. Avoiding high C_trough_ in this setting may prove beneficial for the prevention of nephrotoxicity without compromising treatment efficacy.

## Supplementary Information

**Additional file 1: Table S1. **Comparison of groups divided based on vancomycin AUC_24_/MIC cutoff. **Table S2.** Comparison of groups divided based on vancomycin trough concentration cutoff

## Data Availability

The datasets generated and/or analyzed during the current study are not publicly available due to institutional regulations on restrictions on disclosure of health information, but are available from the corresponding author on reasonable request.

## Code availability

The datasets generated and/or analyzed during the current study are not publicly available due to institutional regulations on restrictions on disclosure of health information, but are available from the corresponding author on reasonable request.
