# Supplementary material for: Pharmacokinetic/pharmacodynamic parameters of vancomycin for predicting clinical outcome of enterococcal bacteremia
Source: BMC Infect Dis. 2022 Aug 10;22:686. doi: 10.1186/s12879-022-07668-w (PMC9364583; doi:10.1186/s12879-022-07668-w)
Supplement: Supplementary file 1 — Additional file 1: Table S1. Comparison of groups divided based on vancomycin AUC24/MIC cutoff. Table S2. Comparison of groups divided based on vancomycin trough concentration cutoff [file 12879_2022_7668_MOESM1_ESM.docx]

Table S1. Comparison of groups divided based on vancomycin AUC_24_/MIC cutoff

|  | **AUC_24_/MIC < 504**  **(n=22)** | **AUC_24_/MIC ≥ 504**  **(n=43)** | ***p*-value** |
| --- | --- | --- | --- |
| **Demographics** |  |  |  |
| Age (mean, SD) | 64 (15) | 58 (13) | 0.107 |
| Male sex | 13 (59.1%) | 33 (76.7%) | 0.139 |
| Body mass index (kg/m^2^) (mean, SD) | 22.9 (2.8) | 21.8 (3.2) | 0.192 |
| **Comorbidities** |  |  |  |
| Diabetes mellitus | 3 (13.6%) | 5 (11.6%) | >0.999 |
| Liver cirrhosis | 0 (0.0%) | 5 (11.6%) | 0.158 |
| Solid organ transplant | 2 (9.1%) | 6 (14.0%) | 0.706 |
| Solid cancer | 8 (36.4%) | 21 (48.8%) | 0.338 |
| Hematologic cancer | 12 (54.5%) | 16 (37.2%) | 0.182 |
| Charlson comorbidity index (mean, SD) | 5 (3, 7) | 5 (3, 7) | 0.989 |
| **Source of infection** |  |  |  |
| Intraabdominal infection | 13 (59.1%) | 20 (46.5%) | 0.337 |
| Urinary tract infection | 1 (4.5%) | 3 (7.0%) | >0.999 |
| Primary bacteremia | 6 (27.3%) | 17 (39.5%) | 0.416 |
| Others | 2 (9.1%) | 3 (7.0%) | >0.999 |
| **Severity of infection** |  |  |  |
| Pitt bacteremia score | 0 (0, 1) | 0 (0, 2) | 0.789 |
| ICU admission at the onset of bacteremia | 0 (0.0%) | 2 (4.8%) | 0.548 |
| AKI at the onset of bacteremia | 1 (4.5%) | 7 (16.3%) | 0.248 |
| **Laboratory test results** |  |  |  |
| Neutropenia | 11 (50.0%) | 10 (23.3%) | 0.029 |
| White blood cell count (/mm^3^) | 3540 (60, 13710) | 5830 (610, 12030) | 0.467 |
| C-reactive protein (mg/dL) | 7.3 (5.7, 13.7) | 8.9 (4.5, 12.8) | 0.764 |
| Creatinine clearance (mL/min) | 99.1 (64.2, 139.0) | 92.4 (57.9, 122.8) | 0.446 |
| **Concurrent use of nephrotoxic drugs** |  |  |  |
| Aminoglycosides | 3 (13.6%) | 5 (11.6%) | >0.999 |
| Liposomal amphotericin B | 1 (4.5%) | 4 (9.3%) | 0.655 |
| Furosemide | 14 (63.6%) | 26 (60.5%) | >0.999 |
| Vasopressor | 4 (18.2%) | 10 (23.3%) | 0.757 |
| Radiocontrast | 10 (45.5%) | 25 (58.1%) | 0.332 |
| Non-steroidal anti-inflammatory drugs | 5 (22.7%) | 10 (23.3%) | >0.999 |
| Others | 9 (40.9%) | 15 (34.9%) | 0.634 |
| **Factors related to vancomycin treatment** |  |  |  |
| Use of vancomycin loading dose | 5 (22.7%) | 9 (20.9%) | 0.868 |
| Average AUC_24_ during initial 72 hours (mg/L) | 428 (382, 477) | 593 (542, 676) | <0.001 |
| Average AUC_24_/MIC during initial 72 hours | 408 (291, 454) | 621 (579, 751) |  |
| Average trough concentration (μg/mL) (mean, SD) | 10.82 (3.45) | 15.18 (4.24) | <0.001 |
| Duration of vancomycin treatment (days) | 12 (7, 16) | 9 (7, 12) | 0.140 |
| **Other factors potentially related to prognosis** |  |  |  |
| Bloodstream infection due to other microorganism(s) within a month | 10 (45.5%) | 17 (39.5%) | 0.647 |
| Source control indicated | 8 (36.4%) | 18 (41.9%) | 0.669 |
| Source control performed (n=26) | 4 (50.0%) | 8 (44.4%) | >0.999 |
| Consult with infectious disease expert | 13 (59.1%) | 32 (74.4%) | 0.205 |

Abbreviations: SD, standard deviation; AKI, acute kidney injury; ICU, intensive care unit; AUC_24_, area under the curve during 24 h; MIC, minimum inhibitory concentration

Table S2. Comparison of groups divided based on vancomycin trough concentration cutoff

|  | **Trough < 13.94** μg/mL  **(n=37)** | **Trough ≥ 13.94** μg/mL  **(n=28)** | ***p*-value** |
| --- | --- | --- | --- |
| **Demographics** |  |  |  |
| Age (mean, SD) | 61 (13) | 60 (15) | 0.697 |
| Male sex | 26 (70.3%) | 20 (71.4%) | >0.999 |
| Body mass index (kg/m^2^) (mean, SD) | 22.4 (3.1) | 21.9 (3.1) | 0.532 |
| **Comorbidities** |  |  |  |
| Diabetes mellitus | 4 (10.8%) | 4 (14.3%) | 0.717 |
| Liver cirrhosis | 2 (5.4%) | 3 (10.7%) | 0.644 |
| Solid organ transplant | 5 (13.5%) | 3 (10.7%) | >0.999 |
| Solid cancer | 15 (40.5%) | 14 (50.0%) | 0.447 |
| Hematologic cancer | 17 (45.9%) | 11 (39.3%) | 0.591 |
| Charlson comorbidity index (mean, SD) | 5 (3, 6) | 6 (3, 8) | 0.337 |
| **Source of infection** |  |  |  |
| Intraabdominal infection | 21 (56.8%) | 12 (42.9%) | 0.267 |
| Urinary tract infection | 0 (0.0%) | 4 (14.3%) | 0.030 |
| Primary bacteremia | 15 (40.5%) | 8 (28.6%) | 0.318 |
| Others | 1 (2.7%) | 4 (14.3%) | 0.156 |
| **Severity of infection** |  |  |  |
| Pitt bacteremia score | 0 (0, 1) | 0 (0, 2) | >0.999 |
| ICU admission at the onset of bacteremia | 2 (5.6%) | 0 (0.0%) | 0.502 |
| AKI at the onset of bacteremia | 3 (8.1%) | 5 (17.9%) | 0.275 |
| **Laboratory test results** |  |  |  |
| Neutropenia | 15 (40.5%) | 6 (21.4%) | 0.103 |
| White blood cell count (/mm^3^) | 2730 (250, 9620) | 8050 (650, 13810) | 0.223 |
| C-reactive protein (mg/dL) | 9.6 (5.5, 13.1) | 8.1 (4.4, 12.1) | 0.451 |
| Creatinine clearance (mL/min) | 99.2 (74.9, 137.1) | 80.3 (52.8, 103.2) | 0.057 |
| **Concurrent use of nephrotoxic drugs** |  |  |  |
| Aminoglycosides | 6 (16.2%) | 2 (7.1%) | 0.449 |
| Liposomal amphotericin B | 2 (5.4%) | 3 (10.7%) | 0.426 |
| Furosemide | 20 (54.1%) | 20 (71.4%) | 0.154 |
| Vasopressor | 8 (21.6%) | 6 (21.4%) | >0.999 |
| Radiocontrast | 18 (48.6%) | 17 (60.7%) | 0.334 |
| Non-steroidal anti-inflammatory drugs | 8 (21.6%) | 7 (25.0%) | 0.749 |
| Others | 14 (37.8%) | 10 (35.7%) | >0.999 |
| **Factors related to vancomycin treatment** |  |  |  |
| Use of vancomycin loading dose | 8 (21.6%) | 6 (21.4%) | 0.985 |
| Average AUC_24_ during initial 72 hours (mg/L) | 466 (411, 580) | 622 (552, 703) | <0.001 |
| Average AUC_24_/MIC during initial 72 hours | 498 (404, 593) | 641 (556, 718) | <0.001 |
| Average trough concentration (μg/mL) | 11.15 (8.69, 12.40) | 17.58 (14.98, 18.80) |  |
| Duration of treatment (days) | 12 (8, 14) | 8 (7, 12) | 0.044 |
| **Other factors potentially related to prognosis** |  |  |  |
| Bloodstream infection due to other microorganism(s) within a month | 16 (43.2%) | 11 (39.3%) | 0.749 |
| Source control indicated | 14 (37.8%) | 12 (42.9%) | 0.683 |
| Source control performed (n=26) | 6 (42.9%) | 6 (50.0%) | >0.999 |
| Consult with infectious disease expert | 25 (67.6%) | 20 (71.4%) | 0.738 |

Abbreviations: SD, standard deviation; AKI, acute kidney injury; ICU, intensive care unit; AUC_24_, area under the curve during 24 h; MIC, minimum inhibitory concentration
